# Abnormal Endometrial Receptivity and Oxidative Stress in Polycystic Ovary Syndrome

**DOI:** 10.3389/fphar.2022.904942

**Published:** 2022-07-25

**Authors:** Hongying Shan, Renxin Luo, Xuanying Guo, Rong Li, Zhenhong Ye, Tianliu Peng, Fenting Liu, Zi Yang

**Affiliations:** ^1^ Center for Reproductive Medicine, Department of Obstetrics and Gynecology, Peking University Third Hospital, Beijing, China; ^2^ First Affiliated Hospital, School of Medicine, Shihezi University, Beijing, China

**Keywords:** polycystic ovary syndrome, endometrial receptivity, oxidative stress, molecular mechanism, hyperandrogenemia

## Abstract

Polycystic ovary syndrome (PCOS) is a common endocrine and metabolic disorder in women of childbearing age. Individual heterogeneity is evident, and the prevalence rate ranges between 6 and 15% globally. The prevalence rate of PCOS in Chinese women of childbearing age is 5.6%. The main manifestations are infertility, sparse menstruation, irregular vaginal bleeding, long-term endometrial hyperplasia, and endometrial cancer. PCOS is often associated with hyperandrogenemia, insulin resistance, hyperinsulinemia, obesity, metabolic syndrome, and intestinal flora disorder. Although there have been many studies in the past, the underlying pathophysiological mechanism of the disease is still unclear. Studies have shown that PCOS diseases and related complications are closely related to local oxidative stress imbalance in the endometrium, leading to poor endometrial receptivity and effects on pregnancy. Previous reviews have mainly focused on the abnormal mechanism of ovarian oxidative stress in women with PCOS, while reviews on endometrial receptivity and oxidative stress are relatively insufficient. This study reviews the abnormal cellular and molecular mechanisms of oxidative stress due to comorbidities in women with PCOS, leading to a downregulation of endometrial receptivity.

## Introduction

Polycystic ovary syndrome (PCOS) is a common endocrine and metabolic disorder in women of childbearing age with evident individual heterogeneity and a prevalence rate of between 6 and 15% (Fauser et al., 2012). For Chinese women of childbearing age, the prevalence rate is 5.6% (Li et al., 2013). The main manifestations of PCOS are infertility, oligomenorrhea, irregular vaginal bleeding, long-term endometrial hyperplasia, and endometrial cancer, which are associated with hyperandrogenemia, insulin resistance (IR), hyperinsulinemia, obesity, and metabolic syndrome (MetS). PCOS was first reported by Stein and Leventhal in 1935 and is also known as Stein–Leventhal syndrome. Due to its clinical heterogeneity, the diagnosis of PCOS has always been controversial. The NIH, Rotterdam, AES, and other diagnostic criteria have been developed internationally, but the Rotterdam standard is the most widely used (Rotterdam ESHRE/ASRM-Sponsored PCOS Consensus Workshop Group, 2004). The Rotterdam criteria are as follows: 1. anovulation or sparse ovulation; 2. polycystic ovarian changes, revealed by an ultrasound showing that one or both ovaries have ≥12 follicles with a 2–9-mm diameter and/or an ovarian volume ≥10 ml; and 3. if two of the three are present, the patient can be diagnosed with clinical hyperandrogenemia and/or biochemical hyperandrogenemia manifestations. PCOS can be diagnosed if two of the three items are met, but other diseases causing hyperandrogenism, hyperprolactinemia, and abnormal thyroid function should be excluded.

The underlying cause of PCOS has not been identified; however, PCOS has been related to genetic and environmental factors. Women of childbearing age mainly present with ovulation disorders. After lifestyle adjustments are made, and metabolic diseases are corrected; clomiphene, letrozole, tamoxifen, gonadotropins, and other drugs are given to induce ovulation. The ovulation rate is approximately 60–80%, although the clinical pregnancy rate is only approximately 35–40% (Bansal et al., 2021; Yland et al., 2022). Therefore, the proportions of failed embryo implantations and spontaneous abortions in women with PCOS are still relatively high. During assisted reproductive technology (ART), oocytes donated by women with PCOS do not reduce the overall pregnancy success rate compared with those donated by healthy women (Ashkenazi et al., 1995), which strongly suggests that the decreased fertility of women with PCOS is related to a disturbance of the body’s environment, decreased oocyte quality, and decreased endometrial receptivity (ER). ER refers to the ability of the endometrium to allow the embryo to adhere and invade, inducing a series of corresponding changes in the endometrium that enable embryo implantation. This period is also called the “implantation window period” and occurs 5–7 days after ovulation. To cope with these changes, precise mechanisms involving numerous molecules and pathways are needed. Two-thirds of clinical repeated implantation failures are caused by insufficient ER (Craciunas et al., 2019), so poor ER has become the primary reason for implantation failure.

Oxidative stress (OS) refers to the excessive production of reactive oxygen species (ROS), an oxidation degree exceeding the scavenging ability of oxides, and an imbalance between the oxidation and antioxidant defense systems. ROS include superoxide anion (O₂-), hydroxyl radical (·OH), and hydrogen peroxide (H₂O₂). There are two types of antioxidant systems in the body: the enzyme antioxidant system and the non-enzymatic antioxidant system. The enzyme antioxidant system includes superoxide dismutase (SOD), catalase (CAT), and glutathione peroxidase (GSH-Px). The non-enzymatic antioxidant system includes vitamin C, vitamin E, glutathione, melatonin, α-lipoic acid, carotenoids, and the trace elements copper, zinc, and selenium.

**FIGURE 1:**
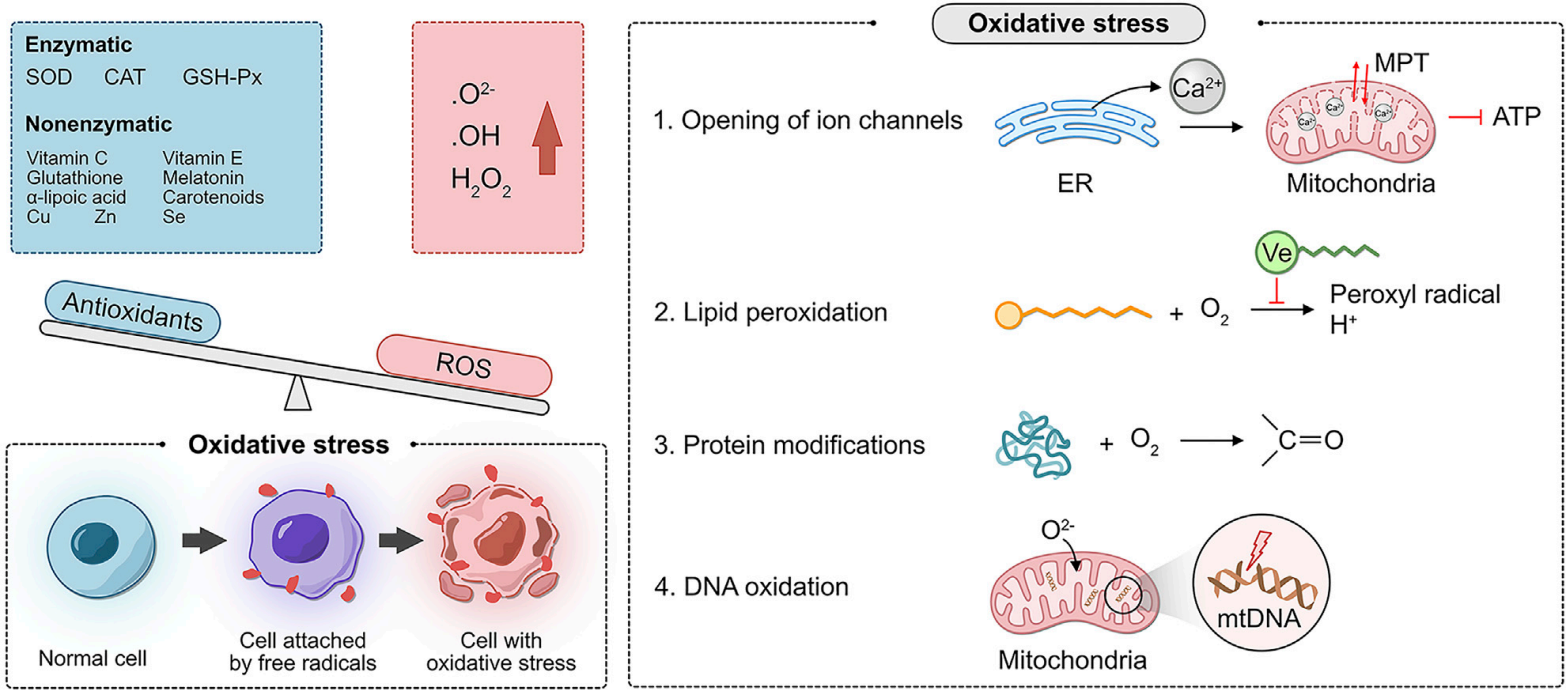
Molecular mechanism of the oxidative stress disorder.

Oxidative phosphorylation (OXPHOS) is fundamental for life. Mitochondria maintain OXPHOS by generating a membrane potential gradient generated by the electron transport chain (ETC) to drive ATP synthesis. The ETC is the main source of ROS generation (Chakrabarty and Chandel, 2021). Moderate levels of ROS stimulate cell growth and proliferation and are necessary for maintaining physiological functions (Mittler, 2017). Chronic ROS accumulation can occur in various cell types, such as vascular endothelial cells, oocytes, endometrial epithelial cells, and stromal cells. Internal signaling pathways directly or indirectly induce cell and tissue damage (i.e., damage to DNA, lipid membranes, and proteins), leading to various female reproductive system disorders (Zhang et al., 2019). These deleterious attacks are mediated by the following more specialized mechanisms (Agarwal et al., 2012):

1. Opening of ion channels: excess ROS leads to Ca^2+^ release from the endoplasmic reticulum, resulting in increased mitochondrial permeability. As a result, the mitochondrial membrane potential is altered, affecting the production of ATP.
2. Lipid peroxidation: this alteration is common in regions, where unsaturated fatty acid side chains are present. These side chains react with O_2_ to produce peroxyl radicals, which can acquire H^+^ from another fatty acid, forming a continuous reaction. Due to the lipid solubility and hydrophobic tail of vitamin E, it can break this chain reaction.
3. Modification of proteins: amino acids are targets of oxidative damage, and direct oxidation of side chains can lead to the formation of carbonyl groups.
4. DNA oxidation: mitochondrial DNA is vulnerable to ROS attack because of the lack of histone protection and repair mechanisms of O^2-^ in the ETC (Figure 1).

OS is closely related to the PCOS onset. Compared with the level in a healthy control group, the OS level in women with PCOS was significantly increased (Table 1). The aforementioned literature suggests that the imbalance of OS in the body is associated with adverse pregnancy outcomes in patients with PCOS. Further studies have shown that ER in women with PCOS is significantly reduced, which is closely related to IR, hyperandrogenemia, metabolic disorders, and intestinal flora imbalance, resulting in an imbalance of endometrial OS (Luo et al., 2017). However, previous reviews have mainly focused on the imbalance of OS and oocyte development, the follicular developmental microenvironment, and embryonic development in PCOS patients, and little attention has been given to research on ER. Therefore, the next section will review the decrease in ER and the cellular and molecular mechanisms of OS in PCOS women with distinct metabolic profiles.

**TABLE 1:** Oxidative stress (OS) markers of women with polycystic ovary syndrome (PCOS).

Biomarkers evaluating the OS level	Location and source	OS levels in PCOS patients (Althobiti et al., 2020)	Reference
p47^phox^↑	Plasma MNC	Normal lean controls	González et al. (2006)
ROS↑; p47^phox^ ↑; TBARS↑	Plasma MNC	Healthy women were treated with 130 mg of dehydroepiandrosterone (DHEA) or placebo (*n* = 8 each)	González et al. (2012a)
TNF-α↑; IL-6 ↑; IL-1β↑	Plasma MNC	Healthy normal lean controls	González et al. (2020)
Prolidase activities↑, TOS↑, OSI↑; TAS↓	Plasma	Healthy normal controls	Hilali et al. (2013)
Fetuin-A↑; Lipid fractions→; MDA→; MPO→; GSH↓; SOD→	Serum	Healthy control women	Enli et al. (2013)
MDA→OR↑; TAC→; CoQ10 ↓	Plasma MNC	Normal weight PCOS	Perovic Blagojevic et al. (2021)
TOS↑; AOPPs↑; MDA↓; PAB↑; TAS↓; SOD↓; PON1→	Plasma	Non-obese women with PCOS	Amato et al. (2003)
SOD↓; MDA↑; SOD↓; TAA↓; VC→; VE↓; RET↓	Plasma	Healthy control women	Wang et al. (2019)

TOS, total oxidant status; OSI, oxidative stress index; TAS, total antioxidant status; MDA, malondialdehyde; MPO, myeloperoxidase; GSH, glutathione; SOD, superoxide dismutase; TAC, total antioxidant capacity; PAB, pro-oxidant–antioxidant balance; PON1, paraoxonase 1; AOPPs, advanced oxidation protein products; TAA, total antioxidant activity; VC, vitamin C; VE, vitamin E; RET, retinol

## Insulin Resistance and Oxidative Stress

Insulin is a protein hormone secreted by pancreatic *β* cells. Insulin binds to the insulin receptor (INSR) and is transferred from intracellular vesicles to the plasma membrane through glucose transporter-4 (GLUT-4) to achieve glucose uptake and utilization. Inhibiting glycogen degradation and gluconeogenesis play a vital role in glucose homeostasis. The anti-OS effect of insulin has been confirmed *in vitro*, *in vivo*, and by physiological and pharmacological studies (Lin et al., 2015; Ruegsegger et al., 2018). Approximately 75% of lean women and 95% of obese women with PCOS have IR (Stepto et al., 2013), and IR and hyperinsulinemia are the core mechanisms of PCOS. In addition, women with PCOS exhibit localized IR, and endometrial IR is mainly due to impaired key molecules in the endometrial insulin pathway and disturbed signaling, leading to decreased glucose uptake (Palomba et al., 2021). These key molecules and pathways include endometrial GLUT-4 (Cabrera-Cruz et al., 2020), INSR substrate phosphorylation downregulation, and PI3K/Akt pathway abnormalities. Local endometrial IR is associated with hyperandrogenemia, obesity, and chronic low-grade inflammation and is particularly closely related to OS imbalance, leading to an upregulation of the OS induced by ROS and pregnancy impairment. The infusion of physiological insulin into obese women with PCOS inhibits ROS generation and the activation of NF-κB (Dandona et al., 2001). Animal experiments (Hu et al., 2019) have shown that compared with that of a control group, IR in mice was related to changes in uterine morphology and the abnormal expression of genes related to endometrial decidualization, placenta formation, angiogenesis, and insulin signaling. Moreover, murine IR is associated with endometrial mitochondrial function and homeostasis (i.e., mitochondrial DNA copy number and the expression of genes responsible for mitochondrial fusion, division, biogenesis, and phagocytosis) changes and the inhibition of oxidative and antioxidant defenses (i.e., reactive oxygen species, nuclear factor erythroid-2 related factor 2 (Nrf2) signaling, and antioxidation). However, most research has been performed on rats, and it is unclear whether these insights can be transferred to long-lived mammals such as humans. The interactive network of the OS response to hyperandrogenemia and IR suggests that both induce mitochondrial-mediated damage in the uterus during pregnancy and lead to the unbalanced relationship between oxidative and antioxidative stress.

The mechanistic target of rapamycin (mTOR) is a member of the phosphatidylinositol 3-kinase-related kinase superfamily. As an essential molecule for signal transduction, cell proliferation, growth, differentiation, and apoptosis, mTOR plays a vital role in mammalian growth control (Somers and Paul, 2015; Wataya-Kaneda, 2015; Vahidnezhad et al., 2016). The expression of molecules related to the mTOR signaling pathway is closely related to ER (Wollenhaupt et al., 2013; Mahdi et al., 2015). Previous studies have shown that an injection of the mTOR inhibitor rapamycin reduces ER (Li et al., 2017). In maternal hyperinsulinemic mice, phosphorylated mTOR (p-mTOR) and p-p70S6K protein expression are reduced, and the expression of genes related to uterine receptivity, namely, Esr1, Pgr, Hoxa10, and Esr2, is deregulated. After insulin treatment, the impaired ER is reversed (Li et al., 2017). Likewise, in women with PCOS and IR, the use of the insulin sensitizer metformin may directly act on the endometrium, reducing IR by increasing the GLUT4 expression and thereby indirectly restoring endometrial function (Supplementary Figure S1). However, these studies either have a small sample size, or the mechanism research is not in-depth enough. More large-sample prospective randomized studies or more in-depth direct mechanism studies are urgently needed. The aforementioned studies show that ER dysfunction in women with PCOS and IR is closely related to the local OS imbalance in the endometrium.

## Hyperandrogenemia and Oxidative Stress

Hyperandrogenemia (HA) is characterized by elevated testosterone levels in women, leading to clinical manifestations such as acne, hirsutism, and alopecia. Androgens are primarily produced by the ovaries, adrenal glands, and peripheral organs (skin and liver). Among them, the ovaries are the main source of androgens (Rosas et al., 2016). The mechanism of the ovarian origin of HA is as follows (Supplementary Figure S2):

1 Abnormal neurotransmitter-related synthase and receptors in the central nervous system lead to abnormal secretion of the neurotransmitter kisspeptin (Varikasuvu et al., 2019; Skorupskaite et al., 2020) and an increase in the frequency of hypothalamic GnRH release and consequently the production of luteinizing hormone (LH). As the frequency and quantity of kisspeptin release are increased, follicular membrane cells are stimulated, and excessive androgen is secreted.
2 Women with PCOS have a variety of androgen synthase abnormalities, such as the increased activity of 3β-hydroxysteroid dehydrogenase (HSD3β), 17α-hydroxylase (CYP17), cytochrome P450 side-chain lyase (CYPscc), and steroidogenic acute regulatory protein (StAR) overexpression, as well as reduced 21-hydroxylase activity. The impairment of the 21-hydroxylase activity blocks the conversion of 17α-hydroxyprogesterone, which is a precursor hormone and, thus, its entry into the androgen synthesis pathway, leading to increased androgen synthesis.

HA is a typical feature of PCOS. 80% of women with PCOS have elevated serum androgen levels (Li et al., 2013). Clinically, total serum testosterone, androstenedione, dehydroepiandrosterone, and dehydroepiandrosterone sulfate are tested. Most studies find that serum-free testosterone may be a more sensitive indicator for detecting hyperandrogenic diseases (Azziz et al., 2009). In addition, the OS imbalance and inflammatory activation in women with PCOS contribute to the continuous occurrence of HA in PCOS, which may change the expression of proteins related to endometrial development and embryo implantation (Rahman et al., 2018; Mokhtar et al., 2020), thereby impairing the receptivity of the endometrium. Furthermore, women with PCOS and HA have high-density lipoprotein (HDL) antioxidant/anti-inflammatory damage, and the decrease in plasma sex hormone-binding globulin (SHBG) levels is an important cause of HA in PCOS (Sun et al., 2021). Nuclear factor-*κ*B (NF-*κ*B) is a potential crucial mediator of hyperandrogenemia-induced inflammation (González et al., 2012b). WNT5a acts as a pro-inflammatory factor in the ovarian granulosa cells of patients with PCOS. Upregulated expression of WNT5a in PCOS primarily increases inflammation and OS through the PI3K/AKT/NF-κB signaling pathway. Moreover, inducible pro-inflammatory cytokines may further enhance the NF-κB-dependent regulation of the WNT5a expression (Zhao et al., 2015). In addition, a cross-sectional study from China showed that OS inhibits the SHBG expression and secretion by downregulating hepatic nuclear factor-4a (HNF-4a) *in vitro*, which may be an important factor in promoting HA in PCOS (Sun et al., 2021). But some of these studies only compared weight-matched controls, ignoring the fact that a large proportion of polycystic ovary syndrome is overweight. These prospective studies provide us with strong evidence that HA is closely related to OS and is an important reason for decreasing endometrial receptivity in women with PCOS.

## Metabolic Disorders and Oxidative Stress

MetS is a complex group of metabolic disorders in which the metabolism of proteins, fats, carbohydrates, and other substances in the human body is disturbed. The central aspects of MetS are obesity and IR, with the main manifestation being obesity, especially central obesity. MetS is a global public health problem. According to statistics, the prevalence of MetS is approximately 20–30% (LeRoith, 2008). The diagnosis of MetS is made when three of the following five criteria are met (Supplementary Figure S3) (Alberti et al., 2009):

1. Central obesity or abdominal obesity: men with MetS have a waist circumference of 90 cm or more, and women with MetS have a waist circumference of 85 cm or more.
2. Elevated fasting blood triglycerides (>150 mg/dl or drug therapy for elevated triglycerides).
3. Decreased high-density lipoprotein (HDL) cholesterol (<40 mg/dl or pharmacological treatment for decreased HDL cholesterol).
4. Elevated blood pressure (systolic pressure >130 mmHg and/or diastolic pressure >85 mmHg, or hypertension has been diagnosed and treatment has been started).
5. Elevated fasting blood glucose (>100 mg/dl or pharmacological treatment for elevated blood glucose).

MetS is characterized by an abnormal increase in multiple body indicators, especially plasma-free fatty acids (FFAs) (Saklayen, 2018). Elevated plasma FFAs cause a progressive decline in insulin secretion by promoting pancreatic β-cell death through increased production of ROS, which activates the generation of ROS (Inoguchi et al., 2000). Compared with women in a control group, women with or without MetS combined with PCOS had a lower antioxidant capacity, and the OS level in the combined MetS group was higher. Similarly, the pathogenetic analysis showed that compared with the level in a control group, the average MDA level in women with PCOS was increased (Pei et al., 2021). In contrast, the exposure of adipose tissues to OS under MetS circumstances, leading to reduced secretion of adiponectin, which exerts anti-inflammatory effects and increased secretion of inflammatory cytokines (Soares et al., 2005; Otani, 2011), led to a compromised insulin signaling pathway through the induction of insulin receptor phosphorylation and the exacerbation of GLUT4 translocation and gene transcription (Bloch-Damti and Bashan, 2005).

A study strongly suggested that impaired lipid patterns may lead to OS activation and weakened antioxidant capacity in women with PCOS combined with MetS (Wang et al., 2019). In addition, in the circulation, the level of HDL, which is an antioxidant, is reduced, and the level of LDL that induces OS is increased. The two work together to further activate OS (Holvoet, 2008; Zelzer et al., 2011). These results suggest that women with PCOS with MetS have higher OS levels and lower antioxidant capacity than women with PCOS without MetS. Studies in rodent livers and endothelial cells have shown that fructose can drive OS by increasing triglyceride synthesis and uric acid production (Johnson et al., 2013). Fructose-induced hyperglycemia and fatty liver mouse models suggest that impaired antioxidant defenses contribute to the pro-oxidant environment in the uterus. In C57BL/6 mice fed with a high-fat diet, proliferating cell nuclear antigen (PCNA) expression decreased, and activated B cells (NF-κB) and the nuclear factor κ-light chain enhancer of the peroxisome proliferator-activated receptor gamma (PPAR gamma) signaling pathway increased (Cheng et al., 2018). With the increase in OS, the high-fat diet led to increased endometrial cell apoptosis by increasing the expression of 8-hydroxydeoxyguanosine (8-OHdG) (Heard et al., 2016); compared with the control group, the high-fat diet in the hypercholesterolemia rat model promoted oxidative and inflammatory stress and significantly increased tumor necrosis factor-α (TNF-α) and F4/80 macrophage infiltration (El-Mansi et al., 2019). The aforementioned studies all suggest that the impaired lipid pattern in PCOS patients with MetS can activate ROS through a variety of molecular adjustment mechanisms, resulting in an imbalance in OS in the body, especially in the endometrial region, which in turn affects ER and leads to adverse pregnancy outcomes.

## Disturbance of the Gut Microbiota and Oxidative Stress

The gut microbiota is the most plentiful and functionally critical microflora, encompassing approximately 10^14^ resident microorganisms and commensals within the human intestinal tract (Sun et al., 2012). The intestinal microbiota plays a vital role in metabolism and the immune system (Chung et al., 2012; Krajmalnik-Brown et al., 2012). The composition of the intestinal microbiota of women with PCOS is significantly altered (Liang et al., 2020), which may be related to IR, metabolic abnormalities, and sex hormone abnormalities in women with PCOS. Whole-genome shotgun sequencing showed no significant difference in bacterial alpha diversity between women with PCOS and healthy controls; however, the beta diversity of the PCOS microbiome was significantly reduced, and the community structure among women with PCOS was more even, especially in the obese PCOS group. In addition, compared with that in healthy controls, the abundance of common *Bacteroides vulgatus* in women with PCOS was significantly increased (Lindheim et al., 2017; Liu et al., 2017; Torres et al., 2018; Qi et al., 2019; Zeng et al., 2019). Tremellen et al. proposed the “gut barrier-endotoxemia-inflammation mechanism” hypothesis, which reflects PCOS pathogenesis (Tremellen and Pearce, 2012), where changes in serum markers such as zonulin, intestinal fatty acid-binding protein 2 (FABP2), and bacterial lipopolysaccharide (LPS) are a result of intestinal barrier damage and inflammation (Sturgeon and Fasano, 2016; Stevens et al., 2018). Endotoxemia could play a role in PCOS pathogenesis by initiating the inflammatory activity (Tremellen and Pearce, 2012). LPS produced by intestinal Gram-negative bacteria is a key molecule in the early development of inflammation and metabolic diseases and has an endotoxin effect, inducing macrophage activation (Wellen and Hotamisligil, 2005), which leads to increases in serum TNF-α and interleukin 6 (IL-6) and triggers IR, which in turn leads to excessive ROS production in the gastrointestinal system (Gyuraszova et al., 2017). Moreover, chronic low-grade inflammation promotes HA and obesity in women with PCOS (Franks and Hardy, 2010; Glintborg et al., 2016; Belani et al., 2018), forming a vicious cycle. In short, gut dysbiosis may mediate systemic low-grade inflammation and IR. Changes affecting sex hormones, the gut-brain axis, and other pathological mechanisms are involved in PCOS development. As discussed earlier, IR, HA, and gut microbiota disturbances can form a vicious cycle that mediates OS imbalances, downregulating ER through various mechanisms and resulting in adverse pregnancy outcomes.

In summary, PCOS is a common multifactorial endocrine and metabolic disorder in women of childbearing age with marked individual heterogeneity, and it mainly manifests as infertility, oligomenorrhea, irregular vaginal bleeding, and long-term complications such as endometrial hyperplasia or endometrial cancer. Genetic susceptibility is one of the risk factors for PCOS, and poor lifestyle habits can induce the disease phenotype. Chronic inflammation in PCOS is systemic. In women with PCOS, higher levels of serum inflammation markers, namely, IL-6, IL-16, IL-18, TNF-α, and CRP, are found (Samy et al., 2009; Ebejer and Calleja-Agius, 2013; Long et al., 2017; Blumenfeld, 2019), and the expressions of intercellular adhesion molecule 1 (ICAM-1), TNF-α, and MCP are upregulated (Peng et al., 2016), indicating an OS imbalance (Long et al., 2017). In addition, it should be noted that PCOS patients suffer from inflammation, regardless of body weight. Obesity is only an aggravating factor. IR, HA, metabolic disorders, and gut microbiota imbalance can lead to endometrial OS imbalance through different signaling pathways and downregulation of ER. The changes in the implantation environment caused by inflammatory mediators may cause ER damage in women with PCOS and embryo implantation failure (Ashary et al., 2018). The crosstalk between embryo implantation and the maternal–fetal interface has not been fully elucidated and still needs to be explored in more in-depth, multicenter studies.
