# Supplementary figures and images for: Abnormal Endometrial Receptivity and Oxidative Stress in Polycystic Ovary Syndrome
Source: Front Pharmacol. 2022 Jul 25;13:904942. doi: 10.3389/fphar.2022.904942 (PMC9357999; doi:10.3389/fphar.2022.904942)

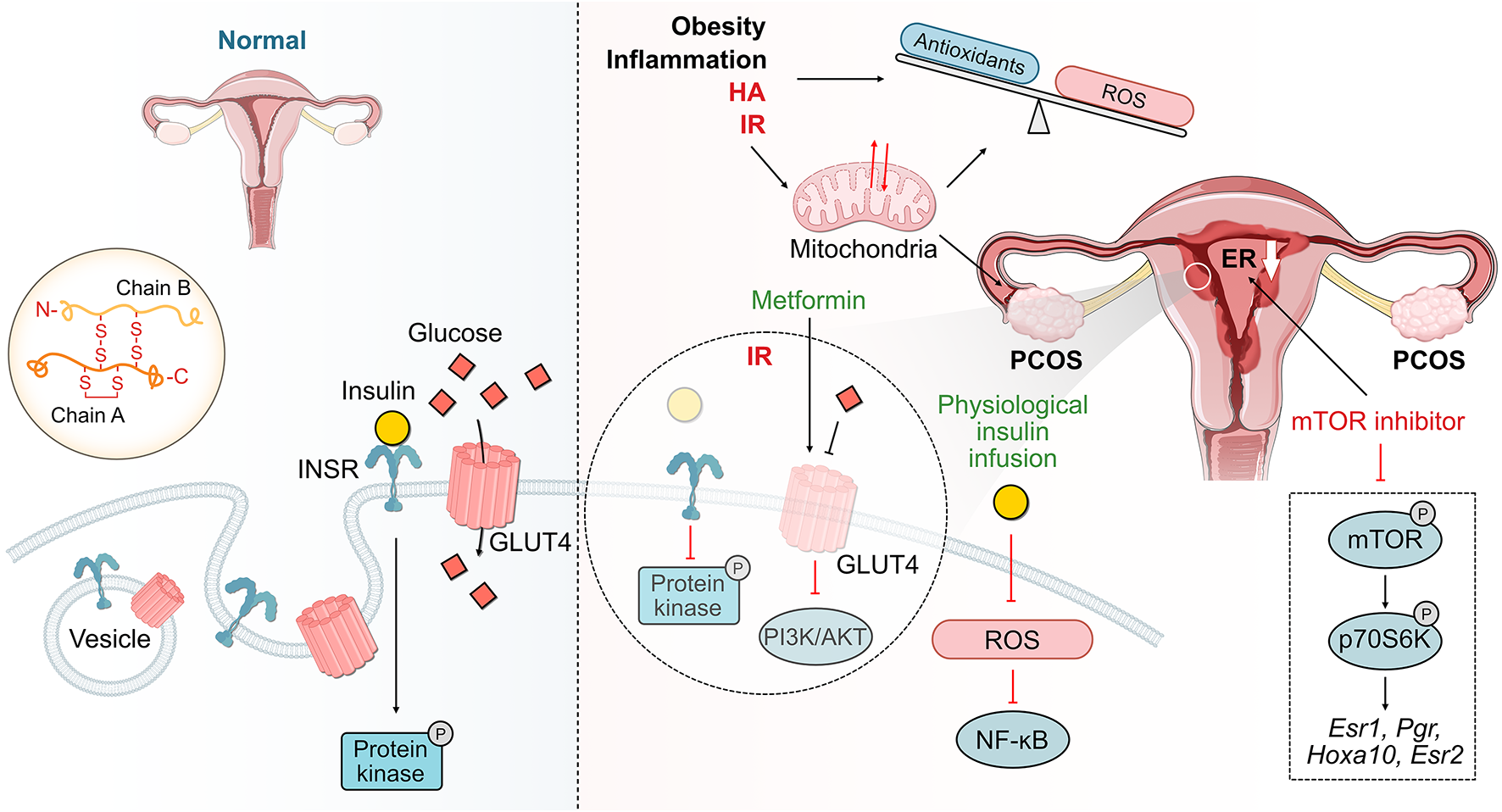

Supplement: Supplementary file 1 [file Image3.TIF]

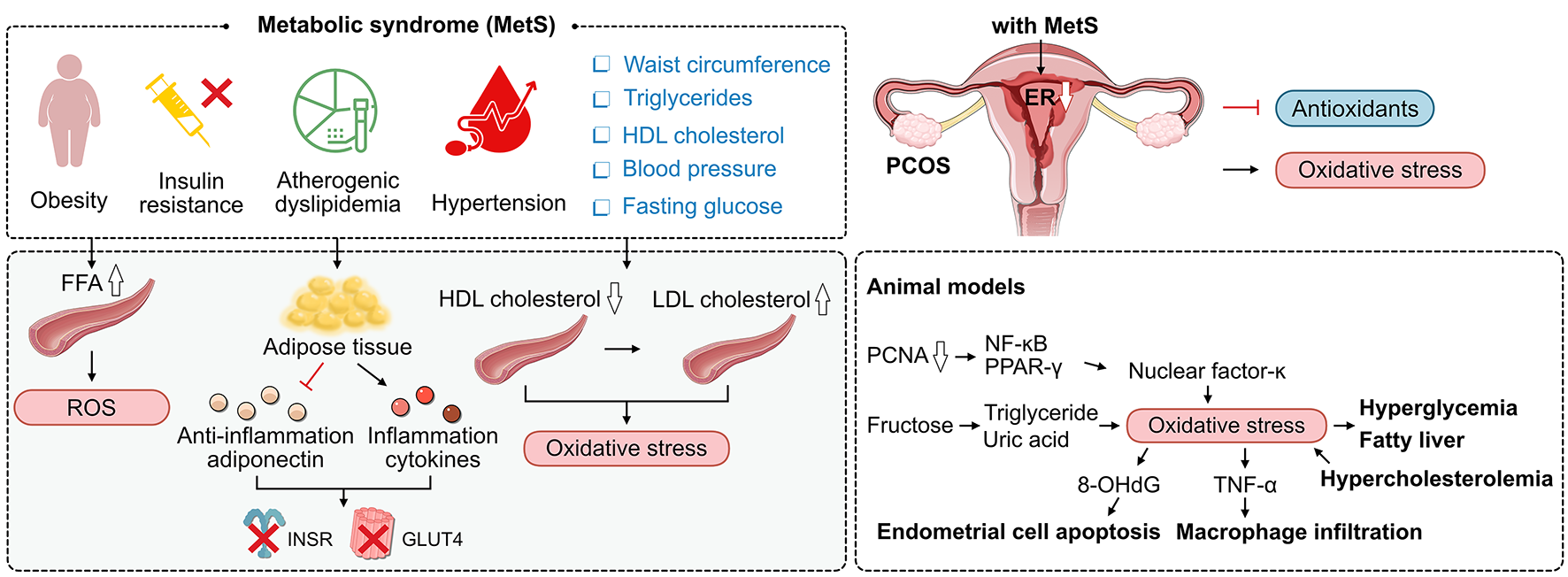

Supplement: Supplementary file 2 [file Image2.TIF]

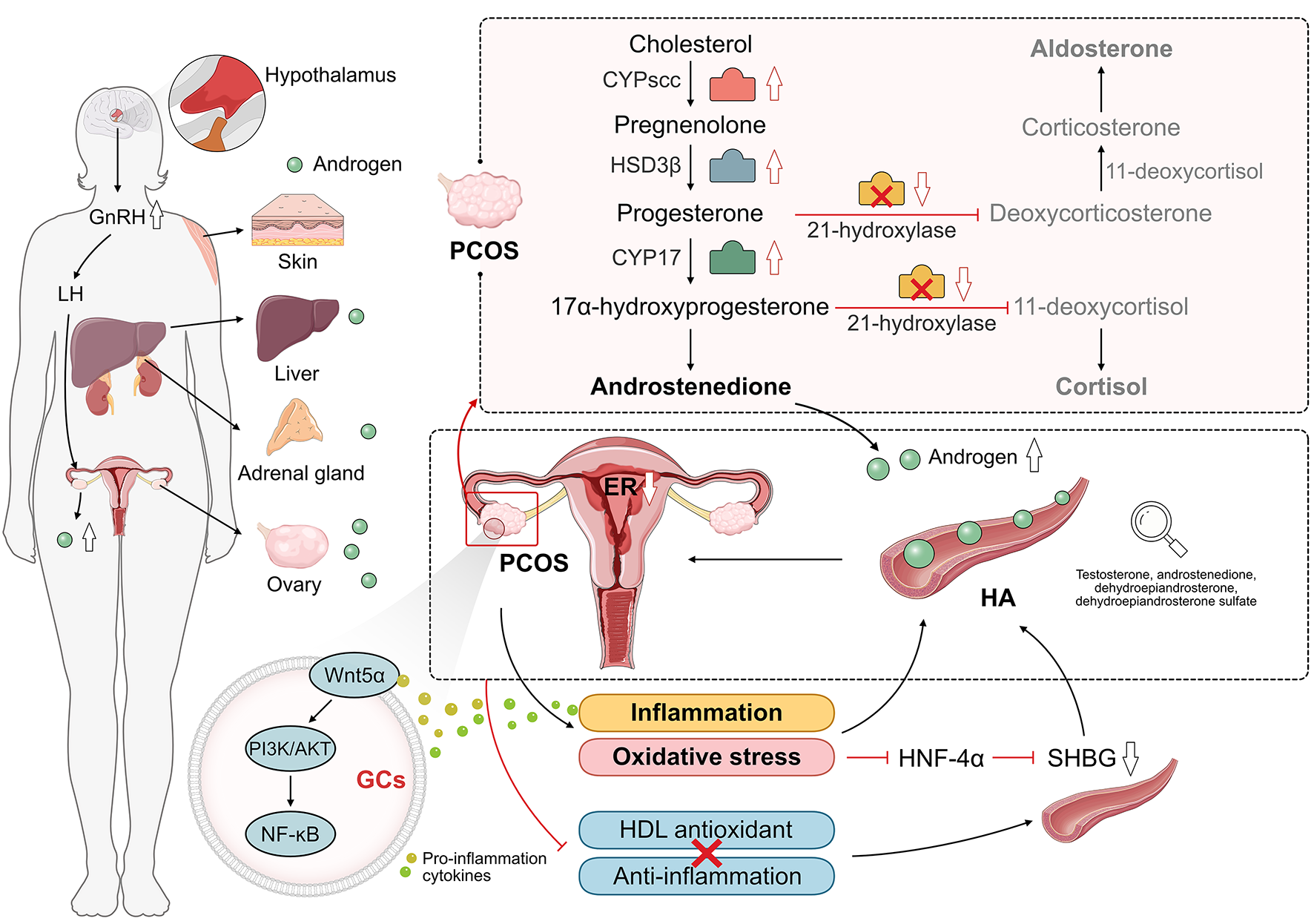

Supplement: Supplementary file 3 [file Image1.TIF]
